# Indoor Particulate Matter Concentration, Water Boiling Time, and Fuel Use of Selected Alternative Cookstoves in a Home-Like Setting in Rural Nepal

**DOI:** 10.3390/ijerph120707558

**Published:** 2015-07-07

**Authors:** Kristen D. Ojo, Sutyajeet I. Soneja, Carolyn G. Scrafford, Subarna K. Khatry, Steven C. LeClerq, William Checkley, Joanne Katz, Patrick N. Breysse, James M. Tielsch

**Affiliations:** 1 Department of International Health, Johns Hopkins Bloomberg School of Public Health, 615 N. Wolfe St., Baltimore, MD 21205, USA; E-Mails: kojo@jhu.edu (K.D.O.); cscrafford@exponent.com (C.G.S.); slecler1@jhu.edu (S.C.L.); wcheckl1@jhmi.edu (W.C.); jkatz1@jhu.edu (J.K.); 2 Department of Environmental Health Sciences, Johns Hopkins Bloomberg School of Public Health, 615 N. Wolfe St., Baltimore, MD 21205, USA; E-Mails: ssoneja1@jhu.edu (S.I.S.); pbreyss1@jhu.edu (P.N.B.); 3 Nepal Nutrition Intervention Project Sarlahi—Harioun, Sarlahi 45804, Nepal; E-Mail: skhatry@wlink.com.np; 4 Division of Pulmonary and Critical Care, Department of Medicine, Johns Hopkins School of Medicine, 1800 Orleans Ave., Suite 9121, Baltimore, MD 21205, USA; 5 Department of Global Health, Milken Institute School of Public Health, George Washington University, 950 New Hampshire Ave., NW Suite 400, Washington, DC 20052, USA

**Keywords:** alternative cookstove performance, airborne particulate concentration, PM, indoor air pollution, biomass fuel use, water boiling test

## Abstract

Alternative cookstoves are designed to improve biomass fuel combustion efficiency to reduce the amount of fuel used and lower emission of air pollutants. The Nepal Cookstove Trial (NCT) studies effects of alternative cookstoves on family health. Our study measured indoor particulate matter concentration (PM_2.5_), boiling time, and fuel use of cookstoves during a water-boiling test in a house-like setting in rural Nepal. Study I was designed to select a stove to be used in the NCT; Study II evaluated stoves used in the NCT. In Study I, mean indoor PM_2.5_ using wood fuel was 4584 μg/m^3^, 1657 μg/m^3^, and 2414 μg/m^3^ for the traditional, alternative mud brick stove (AMBS-I) and Envirofit G-series, respectively. The AMBS-I reduced PM_2.5_ concentration but increased boiling time compared to the traditional stove (*p*-values < 0.001). Unlike AMBS-I, Envirofit G-series did not significantly increase overall fuel consumption. In Phase II, the manufacturer altered Envirofit stove (MAES) and Nepal Nutrition Intervention Project Sarlahi (NNIPS) altered Envirofit stove (NAES), produced lower mean PM_2.5_, 1573 μg/m^3^ and 1341 μg/m^3^, respectively, relative to AMBS-II 3488 μg/m^3^ for wood tests. The liquid propane gas stove had the lowest mean PM_2.5_ concentrations, with measurements indistinguishable from background levels. Results from Study I and II showed significant reduction in PM_2.5_ for all alternative stoves in a controlled setting. In study I, the AMBS-I stove required more fuel than the traditional stove. In contrast, in study II, the MAES and NAES stoves required statistically less fuel than the AMBS-II. Reductions and increases in fuel use should be interpreted with caution because the composition of fuels was not standardized—an issue which may have implications for generalizability of other findings as well. Boiling times for alternative stoves in Study I were significantly longer than the traditional stove—a trade-off that may have implications for acceptability of the stoves among end users. These extended cooking times may increase cumulative exposure during cooking events where emission rates are lower; these differences must be carefully considered in the evaluation of alternative stove designs.

## 1. Introduction

Exposure to indoor air pollution from biomass combustion is a major source of morbidity and mortality worldwide [1,2]. Indoor air pollution from biomass combustion has been associated with increased risks of cardiovascular disease [3], lung cancer [4], stroke [4], chronic obstructive pulmonary disease [4], ischemic heart disease [4], acute lower respiratory infections (ALRI) [4,5,6], low birth weight (LBW) [5,7], stillbirth [5,7], preterm birth [5], stunting [5], cataracts [4], and all-cause mortality [3,5]. A primary source of indoor particulate matter (PM) exposure is the incomplete combustion of biomass fuels for cooking and heating used by approximately half of all people in developing countries [8]. The Global Burden of Disease 2010 project attributed 3.5 million premature deaths in that year to exposure to household air pollution [9]. While biomass fuels are used by less than 5% of the population in most industrialized nations, they are used by 74% of people in Southeast Asia [10]. These fuels are frequently burned indoors in open fires or traditional cookstoves and generate high concentrations of pollutants including PM and carbon monoxide [10,11]. It is well recognized that alternative cookstove designs are needed to reduce the exposure to household air pollutants among individuals who are dependent on biomass burning stoves for cooking and heating.

A number of alternative stove designs have been produced, such as the *plancha mejorada* cookstove used in Guatemala, the *patsari* in Mexico, the *justa* stove used in Honduras [12,13,14], and variations on the rocket stove and gasification stoves [15]. The *justa* and *plancha* stoves both have doors on the firebox, multiple potholes, and chimneys to vent emissions outside the home. In contrast, the rocket style stoves have a single pot hole and a small hole at the base for fuel to be entered [15]. When compared with traditional or open fire stoves, alternative cookstoves have been shown to reduce PM concentrations when tested in actual homes in Guatemala [12,16], Honduras [14], Kenya [17], Mexico [13,18], and China [19]. In Guatemala, researchers found a reduction in mean PM_3.5_ of 83% comparing the traditional stove to the *plancha mejorada* [12]. In Honduras, researchers found a 73% reduction in mean PM_2.5_ comparing the traditional stove to the *justa* [14]. Considerations such as types of meals prepared in a specific region, number of burners a family needs, and the common types of biomass used as fuel are important variables in stove design.

This research aimed to provide data on cookstove performance in a controlled setting that is most similar to the cultural and environmental conditions where the stoves may be used. We conducted two independent but related studies, Study I and Study II, in Sarlahi District, Nepal, which is located in the Terai region of the country. Studies I and II are linked with phases one and two of the Nepal Cookstove Trial (NCT)—a large randomized trial designed to examine the impact of alternative cookstoves on acute lower respiratory infections (ALRI) in children [20]. This manuscript does not report the results of the NCT, but presents results of Studies I and II. Phase one was a modified step-wedge design randomized trial which included six months of morbidity and environmental assessment followed by 12 months where the traditional stove was replaced with an alternative cookstove. When phase one of the Nepal Cookstove Trial was being planned, it was unknown which alternative cookstove would be used to replace the traditional stove.

The goal of Study I, therefore, was to select the most efficient and locally acceptable cookstove that could also be produced reliably on a large scale for phase one of the NCT. After looking at the results from Study I, presented in this manuscript, it was decided that the Envirofit G-series stove would be the best one to use in phase one of the NCT. When compared to the traditional stove, it provided similar reductions in PM_2.5_ as the alternative mud brick stove version I (AMBS-I) and could be produced more reliably on a large scale. Phase two was a randomized trial in households who had received an alternative cookstove as part of phase one. In this new phase, however, households were either randomized to receive a modified Envirofit G-series stove or a liquid propane gas (LPG) stove. During phase one, there was a concern that the Envirofit G-series stove was not reducing PM_2.5_ as much as expected, and so it was decided that another commercially available stove, the LPG would be tested alongside the Envirofit G-series stove in phase two. Before the start of phase two, the stove manufacturer, Envirofit, modified the two-pot stove top extension for the G-series stove so that heat could be transferred more readily to the pot openings. While the NNIPS team waited for Envirofit’s modified stove top to be shipped, they created a modified stove top according to Envirofit’s specifications.

The goal of Study II was to evaluate these two variations of the Envirofit G-series stove with the LPG stove that was being used in phase two of the NCT along with a locally produced alternative mud brick stove version II (AMBS-II). Study II provided information on stove performance using a more controlled setting than the measurements taken from individual homes as part of phase two.

Study I compared the alternative stoves to a traditional stove while Study II compared the alternative designs to a locally produced alternative mud brick stove (AMBS-II). In both Study I and Study II, the stoves were tested in a controlled setting, but not a laboratory setting. The controlled setting in both studies was a test house that was similar in construction and design to houses in the area. The controlled setting allowed for standardization of the amount of thermodynamic work each stove performed, while allowing for variation caused by changing wind patterns. Many studies test only wood as a fuel, whereas Study I and II tested wood in addition to other solid fuels such as crop waste and dried dung. Many studies combine PM concentrations when stoves were in use and not in use and do not attempt to quantify PM concentrations exclusively when stoves are in use. Studies I and II provided measurements for when the stoves are in use adjusted for background levels, and thus captured more closely the PM concentrations associated with the stoves.

## 2. Materials and Methods

As mentioned previously, the purpose of Study I was to choose an alternative stove to be used in phase I of the randomized trial being conducted in Nepal. Potential alternative stoves needed to have at least two burners and a chimney vented to the outside. Products available at the time of study initiation in 2009 included a locally installed mud brick stove and two versions manufactured by Envirofit, Inc. (Fort Collins, CO, USA). Included here are results from the newer stove from Envirofit, the Envirofit G-series stove.

### 2.1. Testing Locations

**Figure 1:**
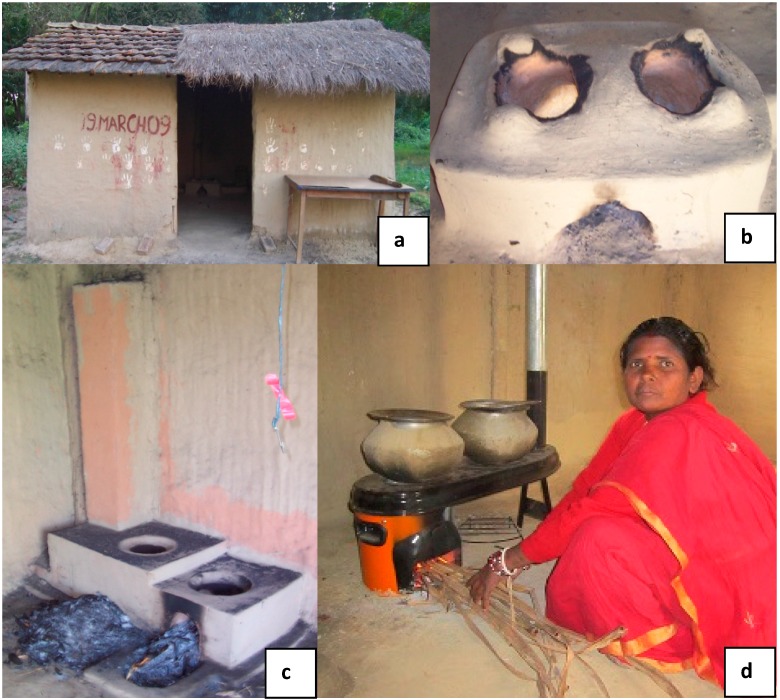
Study I photos of testing house and stoves used in water boiling test study in rural Nepal. (**a**) Single room test house, (**b**) traditional stove, (**c**) alternative mud-brick stove (AMBS-1), and (d) Envirofit G-series stove.

For Study I, sampling was conducted in a mock house built to represent a typical house in this region. The house walls were constructed of mud and bamboo (see Figure 1). These walls formed an area 4.2 m long × 3.3 m wide × 1.88 m high. The central beam in the house was 2.42 m high. The house has one opening for a door that is 0.88 m wide × 1.88 m high. The kitchen volume was 29.8 m^3^. The traditional stove used in Study I was located on the floor against the back wall, and the alternative mud brick stove (AMBS-I) and Envirofit G-series stove were located on the floor on the right hand wall, from the perspective of the door.

For Study II, sampling was conducted in a mock house built to represent a typical house in this region, determined from data collected during the parent cookstove trial. The mock house consisted of a 1-room floor plan with 1-window and door, with the ability to close and open these features. Housing material consisted of bamboo with mud, logs, and tree branches, while roof material consisted of half thatch and half tile. House dimensions consisted of length 3.85 m, width 4.65 m, ground to the lowest point of the roof 1.8 m, ground to the apex of the roof 2.7 m, window 0.6 m by 0.6 m (located on the back wall of the house), and door frame 1.28 m width by 1.64 m height (located on the front wall). The kitchen volume was 40.3 m^3^. Both the window and door had a hinged wood-framed metal panel attached to it that allowed for opening/closing according to the prescribed test conditions. All stoves utilized for Study II were located on the floor against the back wall, with the exception of the Liquid Propane Gas (LPG) stove, which was placed on a table, 1 m high at the same location.

### 2.2. Stoves and Fuel Sources

#### 2.2.1. Study I

Stoves tested in Study I included a traditional stove, an AMBS-I, and the Envirofit G-series (model type G3355) with a chimney (See Figure 1). The Envirofit was the only manufactured product that had both 2 burners and a chimney port, and was available in the necessary quantities at the time the trial began. Three types of fuel combinations, used by local families, were evaluated. They were wood, a mixture of wood and dried ruminant dung, and crop waste. The crop waste was composed mostly of sugar cane leaves and stalks and occasionally contained dried corn stalks.

#### 2.2.2. Study II

**Figure 2:**
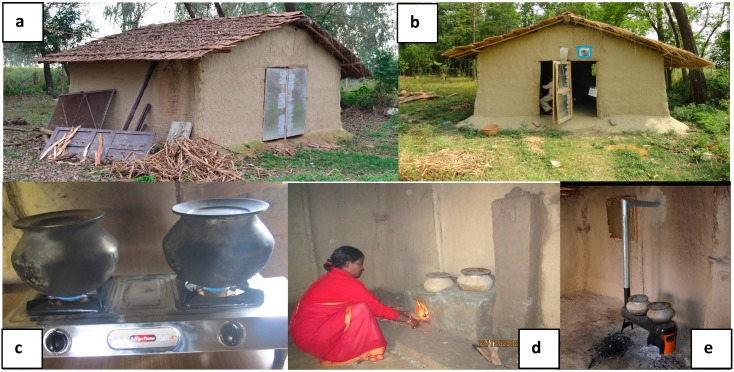
Study II photos of testing house and stoves used in water boiling test study in rural Nepal. (**a**) Single room test house (closed door), (**b**) single room test house (open door), (**c**) liquid propane gas stove (LPG), (**d**) alternative mud brick stove-II (AMBS-II), and (**e**) Envirofit G-series stove (representing MAES and NAES).

Study II evaluated four different stove types, three of which were used in the cookstove trial: the Envirofit G-series with the new modified stove top as produced by the manufacturer (MAES), the Envirofit G-series with a locally modified stove top (NAES), an Alternative Mud Brick stove (AMBS-II), and a Liquid Propane Gas stove (LPG) (see Figure 2). To create the MAES, the manufacturer modified the original G-Series stove top to have an expanded and more centrally located opening for the burner above the firebox so that heat would be transferred more efficiently to both pot openings [21]. To create the NAES, Envirofit G-series stove tops that were in our inventory with the older version of the top were modified by staff from the Nepal Nutrition Intervention Project Sarlahi (NNIPS) to match the newly designed manufacturer altered stove tops. To ensure that the NAES and MAES versions were performing equivalently, both were included in Study II, although there were no known differences aside from whether NNIPS staff or Envirofit had made the modifications. Trials involving the stoves other than the LPG utilized fuel combinations consisting of wood alone and a mixture of wood, dried ruminant dung, and crop waste. These two fuel selections were reported as those most frequently used by families in the NCT [22].

A list of stove abbreviations in Study I and Study II are provided in Table 1.

**Table 1:** Stove Abbreviations for Study I and Study II.

Abbreviation	Explanation of Abbreviation
**Study I**	
Envirofit G-series	Envirofit G-series (model type G3355)
AMBS-I	Alternative Mud Brick Stove (Version I)
**Study II**	
AMBS-II	Alternative Mud Brick Stove (Version II)
MAES	Manufacturer-Altered Envirofit Stove
NAES	NNIPS-Altered Envirofit Stove
LPG	Liquid Propane Gas

### 2.3. Measurement Equipment

Particulate matter concentration was measured with a DataRAM pDR-1000AN (Thermo Scientific, Franklin, MA, USA), hereafter referred to as the pDR. The pDR is a light-scattering photometer that provides real time estimates of PM concentration. It responds best to particles between 0.1 and 10 μm in diameter. In addition, a HOBO U10 Temperature and Humidity Data Logger (Onset Computer Corporation, Pocasset, Bourne, MA, USA), hereafter referred to as the HOBO, was deployed alongside the PM sampling equipment to measure relative humidity (RH). The pDR and HOBO recorded data in 10-s intervals. During the earliest 7 tests in Study I, humidity was not collected with the HOBO every 10-s, but rather was collected at a single time point right before the test began with a wall clock containing a humidity measurement manufactured by Oregon Scientific, Inc. (Tualatin, OR, USA).

#### 2.3.1. Study I Protocol

Study I utilized a modified version of the Water Boiling Test (WBT) 3.0 [23]. Modifications included the use of covered pots (as opposed to uncovered) and a greater volume of water. Four covered pots, each containing 5 L of water, were brought to a boil. The volume of water was modified from the recommended 10 L to 20 L to imitate the water volume necessary for a large meal in Nepal.

The pDR was hung in a stationary location approximately 1 m from the stove and 1 m above the floor. The HOBO hung 0.3 m above the pDR. The pDR ran for 30 min before the stove was lit to provide background measurements. Temperature of the water was measured and fuel was weighed prior to the test beginning. After completion of background measurements, the fire was lit. The pots were placed on the stove according to the following protocol: two pots were placed on the stove (one on each burner); when the first pot boiled, it was removed and the third pot moved to the now vacant stove opening. The next pot that boiled was removed from the stove and the final pot moved to the remaining vacant stove opening. For the AMBS-I and Envirofit G-series, if the last pot to boil was on the cooler burner (the one further away from the main flame), the pot’s position was switched with the other pot that had boiled. The test was finished when the last 5 L of water boiled. The pDR was turned off and the remaining fuel was extinguished in dirt and then post-weighed. Char was not weighed separately from remaining solid fuel. Testing took place from September 2009 to March 2010.

#### 2.3.2. Study II Protocol

In Study II, the same modifications to the Water Boiling Test (WBT) 3.0 were used with the following deviations: 10 L of water were boiled instead of 20 L and upon test completion water was placed upon the fuel with the remnant being removed from the house, dried and post-weighed the following day. In addition, both the pDR and the HOBO were hung together 1 m from the stove and 1.8 m high. Study II incorporated having the window/door open or closed (not conducted in Study I) in order to develop a better understanding of whether our outcomes (mean PM_2.5_ concentration, boil time, and fuel use) would be affected. Testing took place from February to March of 2013. Because of the protocol differences between Study I and Study II, along with the fact that none of the same stoves are tested, results from Study I and II were not compared.

### 2.4. Data Analysis

All statistical analyses for Study I were performed in STATA 10 (STATACorp., College Station, TX, USA). A total of 98 tests were conducted. Of these, three were excluded from analysis for the following reasons: no humidity measurements (n = 2), and interruptions to test (n = 1). Study II statistical analyses were performed in the R statistical computing environment (Version 3.0.0—The R Foundation for Statistical Computing). A total of 48 tests were conducted, with two excluded due to lack of humidity measurements.

Due to high PM_2.5_ concentrations present during cooking in settings utilizing biomass fuels, use of filter-based methods to collect PM can be extremely challenging. However, in order to develop PM_2.5_ measurements equivalent to filter-based collection methods, each 10-s PM measurement was adjusted for RH and converted to a PM_2.5_ gravimetric equivalent using a modified version of a formula from prior literature [24]. The formula was modified so that PM measurements collected at lower RH could be converted to PM_2.5_ (see appendix for the modification). Time to boil was calculated from when the stove was lit until the fire was extinguished. Fuel use was calculated as the difference between pre and post weight measurements. PM concentrations were averaged over the time it took to boil and then adjusted for background PM measurements by subtracting the mean PM concentration during the 30 min prior to test initiation.

Statistical summaries of cookstove performance variables consisting of PM_2.5_ concentration, boiling time, and fuel use were calculated for each stove and fuel type combination. For Study I, multiple linear regression models were used to estimate the effect of the stove type on PM_2.5_ concentration, boiling time, and fuel use. In each of these models, the covariates were stove type, fuel type, and pre-test water temperature. The data for mean PM_2.5_, boiling time, and fuel use were converted to the logarithmic scale to improve normality. The formula for the multiple linear regression model explaining PM_2.5_ in Study I is presented in Equation (1) below: 

(1) $$\text{ln}\left(\text{Y}\right)={\text{β}}_{0}+{\text{β}}_{1}S_{1}+{\text{β}}_{2}F_{2}+{\text{β}}_{3}W_{3}+\;\text{ε}$$

Y is mean PM_2.5_ concentration during cooking adjusted for background. Variables are defined as: *S*_1_ is stove type, *F*_2_ is fuel type, and *W*_3_ is a continuous variable for starting water temperature, and error term ε is independent normal (0, σ_e_^2^). Dummy variables were created for the following stove types: AMBS-I and Envirofit G-series (not shown in the formula). Dummy variables were also created for the following fuel types: wood/dung and crop waste (not shown in formula). The traditional stove and wood fuel were the reference variables for stove type and fuel type, respectively. Identical formulas could be written explaining boiling time or fuel use by replacing Y with these constructs.

Summary statistics for Study II were calculated in the same manner. It should be noted that when including fuel type and window/door status (open *vs.* closed) the sample sizes became small; n ≤ 3 for non-LPG stoves and n = 6 for LPG. For Study II, multiple linear regression models were used to estimate the effect of the stove type on PM_2.5_ concentration, boiling time, and fuel use. For models examining PM_2.5_ concentration, LPG stove data was excluded (see discussion). For models examining PM_2.5_ concentration, boil time, and fuel usage, the covariates were stove type, fuel type, and window/door status being open or closed. LPG stove data was also excluded in models examining fuel use since LPG fuel usage was not measured. No models in Study II adjusted for water temperature, as these data were not collected during Study II. Comparisons for performance measures were made relative to the AMBS-II, with natural log adjusted values for mean PM_2.5_, boil time, and fuel use incorporated into the regression analysis. The formula for the multiple linear regression model explaining PM_2.5_ in Study II is presented in Equation (2) below: 

(2) $$\text{ln}\left(\text{Y}\right)={\text{β}}_{0}+{\text{β}}_{1}S_{1}+{\text{β}}_{2}F_{2}+{\text{β}}_{3}WD_{3}+\text{ε}$$

Y is mean PM_2.5_ concentration during cooking adjusted for background. Variables are defined as: *S*_1_ is stove type, *F*_2_ is fuel type, and *WD*_3_ is open/closed status of the door/window with closed status as the reference and the error term ε is independent normal (0, σ_e_^2^). Dummy variables were created for the following stove types: NAES and MAES (not shown in the formula). Dummy variables were also created for the following fuel types: wood/dung/crop waste (not shown in formula). The AMBS-II and wood fuel were the reference variables for stove type and fuel type, respectively. An identical formula could be written explaining fuel use by replacing Y with this construct. To create a formula for boiling time, one would replace Y with this construct and then add in a dummy variable for the LPG stove for stove type to the existing formula.

## 3. Results

### 3.1. Study I

A total of 95 tests were analyzed in Study I. Comparisons of mean PM_2.5_ concentrations, boiling time, and fuel use are shown in Table 2 and Table 3. Median PM_2.5_ concentrations have been added because the data were not normally distributed. For the tests summarized in Table 2, background measurements have been subtracted from each test’s PM_2.5_ concentration to yield the PM_2.5_ concentration attributable to cooking.

### 3.2. PM Concentration

Both alternative stoves, the AMBS-I and Envirofit G-series, reduced mean PM_2.5_ relative to the traditional stove. PM_2.5_ concentrations among tests using wood for fuel were 1657 μg/m^3^ for AMBS-I, 2414 μg/m^3^ for the Envirofit G-series, and 4584 μg/m^3^ for the traditional stove (see Table 2). Mean PM_2.5_ concentrations using wood and dung ranged from 1317 μg/m^3^ for AMBS-I to 5799 μg/m^3^ for the traditional stove. Crop waste produced the highest mean PM_2.5_ concentrations in comparison with other fuel types; 5442 μg/m^3^ for AMBS-I, 4945 μg/m^3^ for Envirofit G-series, and 9766 μg/m^3^ for the traditional stove. Medians were also calculated. Median PM_2.5_ concentrations among tests using wood for fuel ranged from 1058 μg/m^3^ for AMBS-I to 3294 μg/m^3^ for the traditional stove. Median PM_2.5_ concentrations using wood and dung ranged from 938 μg/m^3^ for AMBS-I to 4220 μg/m^3^ for the traditional stove. Median PM_2.5_ concentrations using crop waste ranged from 4945 μg/m^3^ for the Envirofit G-series to 6299 μg/m^3^ for the traditional stove. In an analysis that controlled for fuel type and starting water temperature (see Table 3), both alternative stoves showed a significant reduction in mean PM_2.5_ concentration as compared to the traditional stove. AMBS-I showed a mean reduction of 60% (*p* < 0.001) in mean PM_2.5_ followed by the Envirofit G-series stove with a 50% (*p* < 0.001) reduction.

**Table 2:** Summary of cookstove performance in Study I comparing two alternative cookstoves to a traditional stove in a home setting in rural Nepal.

Measure	Fuel	WBT or *Background*	Traditional		n	AMBS-I		n	Envirofit G-Series		n
			Mean (SD) ^a^	Median (IQR) ^b^		Mean (SD) ^a^	Median (IQR) ^b^		Mean (SD) ^a^	Median (IQR) ^b^	
**PM_2.5_ Concentration (μg/m^3^)**	Wood	WBT ^c^	4584 (2769)	3294 (2325–5137)	23	1657 (859)	1058 (513–1655)	12	2414 (1191)	1818 (1257–2383)	9
		*Background* ^d^	*148 (154)*	*100 (33–204)*		*83 (52)*	*59 (7–103)*		*217 (128)*	*247 (106–299)*	
	Wood & Dung	WBT ^c^	5799 (2621)	4220 (3605–5582)	13	1317 (455)	938 (425–985)	6	3769 (3539)	2115 (1196–2883)	9
		*Background* ^d^	*119 (80)*	*74 (63–182)*		*51 (36)*	*30 (21–73)*		*199 (104)*	*180 (133–231)*	
	Crop Waste	WBT ^c^	9766 (6788)	6299 (3388–14,824)	12	5442 (3241)	4487 (3783–5345)	5	4945 (2476)	3838 (2680–5665)	6
		*Background* ^d^	*172 (118)*	*145 (52–213)*		*120 (133)*	*26 (21–65)*		*295 (185)*	*186 (148–277)*	
**Boiling Time (hours)**	Wood	WBT	0.69 (0.08)		23	1.17 (0.12)		12	1.47 (0.19)		9
	Wood & Dung	WBT	0.74 (0.09)		13	1.17 (0.11)		6	1.66 (0.38)		9
	Crop Waste	WBT	0.80 (0.09)		12	1.79 (0.22)		5	1.67 (0.30)		6
**Fuel Use (kg)**	Wood	WBT	2.35 (0.33)		23	3.13 (0.50)		12	2.97 (0.38)		9
	Wood and Dung	WBT	2.47 (0.48)		13	2.77 (0.44)		6	2.70 (0.48)		9
	Crop Waste	WBT	2.51 (0.46)		12	3.89 (0.58)		5	2.60 (1.09)		6

Abbreviations: WBT is Water Boiling Test; IQR is interquartile range; SD is standard deviation; AMBS-I is alternative mud brick stove (Version I). **^a^** This summary measure was calculated from water boiling test means or background measurement means (See the column “WBT or Background”); **^b^** This summary measure was calculated from water boiling test medians or the background measurement medians (See the column “WBT or Background”); ^c^ For WBT PM_2.5_, the background PM_2.5_ was subtracted from the PM_2.5_ measured during the water boiling test. The summary measure was calculated according to stove and fuel categories listed in the table; ^d^ Background PM_2.5_ was calculated from measurements taken during the 30-min period before the water boiling test began. The summary measure was calculated according to stove and fuel categories listed in the table.

**Table 3:** Multivariate analysis^*^ of cookstove performance in Study I comparing two alternative cookstoves to a traditional stove in a home setting in rural Nepal.

Measure	Traditional	AMBS-I		Envirofit G-Series	
		Mean (95% CI)	*p*-Value	Mean (95% CI)	*p*-Value
**PM_2.5_ Concentration (% Reduction)**		60 (44, 72)	<0.001	50 (28, 65)	<0.001
**Boiling Time (% Increase)**	Reference	86 (72, 101)	<0.001	104 (88, 121)	<0.001
**Fuel Use (% Increase)**		39 (24, 55)	<0.001	6 (−5, 19)	0.290

^*^ All results were adjusted for fuel type and starting water temperature.

### 3.3. Boiling Time

The alternative cookstoves had longer boiling times than the traditional stove. AMBS-I required 1.17 to 1.79 h to boil while the Envirofit G-series required 1.47 to 1.67 h to boil, depending on fuel type. The traditional stove had the shortest boiling time, with a mean of less than 1 h regardless of fuel type. Just as the mean PM_2.5_ concentrations were elevated when crop waste was used, the use of crop waste fuel type generally required longer boiling times compared to the other fuels, regardless of stove type. An adjusted analysis of the time required to boil 20 L of water showed that both alternative stoves took significantly longer than the traditional stove. Relative to the traditional stove, the Envirofit G-series stove required a mean increase of 104% more time to boil (*p* < 0.001) compared to the traditional stove, while the AMBS-I took 86% longer (*p* < 0.001).

### 3.4. Fuel Use

The average amount of fuel used to boil 20 L of water in four pots was higher for the alternative stoves than the traditional stove in the unadjusted analysis (see Table 2) for all three fuels. Utilizing wood as the fuel source, AMBS-I required 3.13 kg, Envirofit G-series 2.97 kg, and the traditional stove required only 2.35 kg of fuel. Only AMBS-I had significantly (*p* < 0.001) higher fuel use, 39%, compared to the traditional stove when adjusted for fuel type and starting water temperature. Envirofit G-series required 6% more fuel, but this increase was not significant (*p* = 0.29) when adjusted for fuel type and starting water temperature.

### 3.5. Study II

A total of 46 tests were analyzed for Study II. Comparisons of mean PM_2.5_ concentrations, boiling time, and fuel use are shown in Table 4 and Table 5.

**Table 4:** Summary of cookstove performance in Study II comparing three alternative cookstoves with an improved brick stove in a home setting in rural Nepal.

Measure	Fuel/Window & Door Status	WBT or *Background*	AMBS-II	Median (Range) ^b^ *Background*	n	MAES	Median (Range) ^b^ *Background*	n	NAES		n	LPG	Median (Range) ^b^ *Background*	n
			Mean (SD) ^a^ *Background*			Mean (SD) ^a^ *Background*			Mean (SD) ^a^ *Background*	Median (Range) ^b^ *Background*		Mean (SD) ^a^ *Background*		
**PM_2.5_ Concentration (μg/m^3^)**	Wood/Closed	WBT ^c^	3053 (190)	2829 (2414–3062)	3	1626 (609)	N/A **^e^**	2	2947 (2804)	1502 (614–3727)	3			
		*Background* **^d^**	*80 (77)*	*70 (0–160)*		*32 (40)*	*N/*A **^e^**		*36 (29)*	*29 (0–56)*				
	Wood/Open	WBT ^c^	3488 (1744)	1954 (1664–4323)	3	1573 (433)	1298 (919–1514)	3	1341 (468)	1097 (715–1506)	3			
		*Background* **^d^**	*88 (75)*	*111 (0–137)*		*64 (88)*	*17 (0–169)*		*74 (56)*	*75 (14–125)*				
	Wood, Dung, Crop waste/Closed	WBT ^c^	3645 (1385)	2752 (2200–4443)	3	2107 (76)	N/A **^e^**	2	2339 (488)	1990 (1503–2541)	3			
		*Background* **^d^**	*66 (54)*	*46 (18–99)*		*78 (99)*	*N/A* **^e^**		*5 (4)*	*0 (0–2)*				
	Wood, Dung, Crop waste/Open	WBT ^c^	3798 (2736)	2922 (865–4613)	3	3093 (1992)	1854 (1458–4725)	3	2465 (873)	2376 (1406–2915)	3			
		*Background* **^d^**	*114 (60)*	*62 (40–130)*		*79 (51)*	*36 (0–111)*		*51 (81)*	*1 (0–114)*				
	LPG/Closed	WBT ^c^										113 (138) **^f^**	55 (0–384) **^f^**	6
		*Background* **^d^**										*127 (147)*	*69 (52–95)*	
	LPG/Open	WBT ^c^										109 (60) **^f^**	101 (1–177) **^f^**	6
		*Background* **^d^**										*152 (65)*	*135 (111–151)*	
**Boiling Times (hours)**	Wood/Closed	WBT	0.88 (0.03)		3	0.85 (0.02)		2	0.88 (0.08)		3			
	Wood/Open	WBT	0.90 (0.03)		3	0.81 (0.08)		3	0.88 (0.04)		3			
	Wood, Dung, Crop waste/Closed	WBT	1.00 (0.16)		3	0.89 (0.04)		2	0.96 (0.12)		3			

	Wood, Dung, Crop waste/Open	WBT	0.91 (0.01)		3	0.89 (0.08)		3	0.98 (0.05)		3			

	LPG/Closed	WBT										0.43 (0.02)		6
	LPG/Open	WBT										0.50 (0.06)		6
**Fuel Use (kg)**	Wood/Closed	WBT	1.84 (0.18)		3	1.16 (0.09)		2	1.39 (0.33)		3			
	Wood/Open	WBT	1.77 (0.07)		3	1.36 (0.14)		3	1.34 (0.13)		3			
	Wood, Dung, Crop waste/Closed	WBT	1.32 (0.75)		3	1.58 (0.05)		2	1.45 (0.14)		3			
	Wood, Dung, Crop waste/Open	WBT	1.84 (0.03)		3	1.40 (0.14)		3	1.59 (0.06)		3			

Abbreviations: WBT is Water Boiling Test; IQR is interquartile range; SD is standard deviation; AMBS-II is alternative mud brick stove (Version II); MAES is manufacturer-altered Envirofit stove; NAES is NNIPS-altered Envirofit stove; LPG is liquid propane gas stove. **^a^** This summary measure was calculated from water boiling test means or background measurement means (See the column “WBT or Background”); **^b^** This summary measure was calculated from water boiling test medians or the background measurement medians (See the column “WBT or Background”); **^c^** For WBT PM_2.5_, the background PM_2.5_ was subtracted from the PM_2.5_ measured during the water boiling test. The summary measure was calculated according to stove and fuel categories listed in the table; **^d^** Background PM_2.5_ was calculated from measurements taken during the 30-min period before the water boiling test began. The summary measure was calculated according to stove and fuel categories listed in the table; ^e^ Median not reported due to sample size of only n = 2; **^f^** PM_2.5_ concentration not adjusted for background levels due to resultant negative values.

**Table 5:** Multivariate analysis ^a^ of cookstove performance in Study II comparing three alternative cookstoves to an Improved Mud Brick Stove in a home setting in rural Nepal.

Measure	AMBS-II		MAES			NAES			LPG	
			Mean (95% CI)	*p*-Value		Mean (95% CI)	*p*-Value		Mean (95% CI)	*p*-Value
**PM_2.5_ Concentration (% reduction)**			39 (5, 60)	0.025		39 (8, 60)	0.019		**^b^**	
**Boiling Time (% increase)**	Reference		7 (−0.2, 14)	0.056		−0.2 (−8, 7)	0.968		−49 (−45, −53)	<0.001
**Fuel Use (% decrease)**			25 (17, 33)	<0.001		22 (14, 29)	<0.001			

^a^ All results were adjusted for fuel type and window/door status; ^b^ Resultant concentration was indistinguishable from background levels, therefore actual reduction is unknown.

### 3.6. PM Concentration

The LPG stove had the lowest mean PM_2.5_ concentration for both closed and open window/door status with values indistinguishable from background levels regardless of condition. While these results were reported, they were not adjusted for background levels, as this would have resulted in negative values. The AMBS-II had the highest mean PM_2.5_ concentration during cooking for the majority of fuel types and window/door status (see Table 4), ranging from 3053 μg/m^3^ to 3798 μg/m^3^. Median PM_2.5_ concentration and ranges are presented in Table 4. For AMBS-II, median PM_2.5_ concentration regardless of fuel type or window/door status ranged from 2579 μg/m^3^ to 3606 μg/m^3^. For MAES, median PM_2.5_ concentration was not calculated for the closed window/door status for wood and wood/dung/crop waste fuel types due to small sample sizes (n = 2). LPG median PM_2.5_ concentrations were reported as unadjusted for background due to resulting negative values. In an analysis that controlled for window/door status and fuel type, the MAES and NAES stoves both showed a statistically different (*p* < 0.05) reduction in mean PM_2.5_ concentration of 39% (see Table 5) relative to the AMBS-II.

### 3.7. Boiling Time and Fuel Usage

The LPG stove had the lowest time to boil relative to all other stoves, ranging from 0.43 h to 0.50 h regardless of window/door status condition. An adjusted analysis of the time required to boil 10 L of water, accounting for window/door status, showed that the LPG took significantly less time to boil relative to the AMBS-II with a 49% mean reduction in time (*p* < 0.001). For the same analysis, the MAES stove took 7% less time to boil relative to the AMBS-II. This result was borderline significant (*p* = 0.056). The NAES did not result in a significant reduction in boil time (*p* = 0.97). Fuel usage reduction for the NAES and MAES stove was 25% and 22%, respectively, both statistically different (*p* < 0.001) relative to the AMBS-II.

## 4. Discussion

### 4.1. PM Concentration

Study I showed that AMBS-I and the Envirofit G-series stoves reduced mean PM_2.5_ by 60% (*p* < 0.001) and 50% (*p* < 0.001), respectively, compared to the traditional stove. Likewise, Study II showed that the MAES and NAES models of the Envirofit stove significantly (*p* < 0.05) reduced indoor mean PM_2.5_ concentration by 39% each, relative to AMBS-II. Prior studies examining PM_2.5_ concentrations from both traditional and alternative stoves have also reported reductions with use of alternative stoves—82% for the *plancha* measured over 22 h [25], 73% for the *justa* measured over 8 h [14], 65% for the *patsari* measured over 48 h [18], and 62% for the *plancha* measured over 48 h [16]. The PM_2.5_ reductions for the *plancha* [25] and *justa* [14] were greater than those observed in our study for the alternative stoves. The PM_2.5_ reductions for the *patsari* [18] and the *plancha* [14], both of which were measured over 48 h, had similar reductions to the AMBS-I, which was 60% (see Table 3). Reductions in PM_2.5_ found in the literature include cooking and non-cooking time averaged over an 8 to 48 h period, whereas our study only included PM_2.5_ measured during boiling time while the fuel was burning.

Concentrations found in Study I and II are similar to findings in studies measuring indoor PM_2.5_ concentration during cooking periods. In Study I, we observed mean PM_2.5_ concentrations between 1317 and 5442 μg/m^3^ for the AMBS-I and Envirofit G-series cookstoves (see Table 2). In Study II, we observed mean PM_2.5_ concentrations between 1341 and 3798 μg/m^3^ for the MAES, NAES, and AMBS-II cookstoves (see Table 4). Other studies have shown PM_2.5_ concentrations during cooking of 2740 μg/m^3^ for wood burning stoves with no ventilation [26] and 5310 μg/m^3^ for open fire stoves [27].

The mean background concentrations of PM_2.5_ measured during the 30 min before a test began were quite high, with the majority of results greater than 50 μg/m^3^ (see Table 2 and Table 4). In some cases in Study I, the background was over 200 μg/m^3^. WHO guidelines recommend that the mean PM_2.5_ for a 24-h period of time be no more than 25 μg/m^3^ [28]. Although, we did not measure PM_2.5_ for 24 h, the ambient PM_2.5_ values in this region, even in absence of any cooking in the test house, are high in relationship to WHO standards. The high levels of ambient PM_2.5_ might be attributable to the use of biofuels in homes across the community.

Study I and II had tests with high standard deviations, and Study II had some tests where the mean PM was higher when the door and window were open as opposed to closed. In Study I, the traditional stove with wood for fuel had a mean (SD) PM_2.5_ of 4584 μg/m^3^ (2769 μg/m^3^). In Study II, the AMBS-II stove with wood for fuel with a closed window and door had a mean (SD) PM_2.5_ of 3053 μg/m^3^ (190 μg/m^3^) compared with the same stove and fuel with an open window and door that had a mean (SD) PM_2.5_ of 3488 μg/m^3^ (1744 μg/m^3^). Higher PM_2.5_ concentrations when the window was open as opposed to closed and high standard deviations may be due to variable wind patterns. Smoke from the cookstoves may have been blown by the wind in such a way that it made more contact with the pDR causing PM_2.5_ levels to rise. In contrast, the opposite could have occurred where wind might have been blowing the smoke away from the pDR, causing PM_2.5_ levels to decrease. We did not assess meteorological variables, however, so cannot say for certain that wind patterns are responsible for the cases where there is higher mean PM_2.5_ when the window is open or in cases where there is high standard deviation.

Of all the stoves tested in Study II, the LPG stove had the lowest mean PM_2.5_ concentration for both closed and open window/door status with values indistinguishable from background levels ranging from 43 to 423 μg/m^3^ regardless of condition. LPG stoves have been shown to produce levels of PM_2.5_ orders of magnitude lower than stoves using biomass fuels [29,30]. In our study the contribution to PM_2.5_ was negligible, thus resulting in mean PM_2.5_ being indistinguishable from background levels.

### 4.2. Boiling Time and Fuel Usage

In Study I, boiling times were significantly longer for alternative stoves, taking almost twice the time to complete the water boil test compared to the traditional stove regardless of fuel type. The issue of alternative stoves requiring longer cooking times has been reported previously [18,31,32]. In Guatemala, the traditional open stove took 21% less time to boil water than the *plancha* (*p* < 0.05) [31]. In Mexico, researchers reported qualitatively that the open fires could heat larger quantities of liquids more quickly than the *patsari* stove [18].

In contrast, in Study II the alternative stove, NAES, did not show a statistically significant difference in time to boil relative to AMBS-II, while MAES showed a difference that was borderline significant (*p* = 0.056). Not surprisingly, the LPG stove, with its consistent and uniform delivery of heat, had a much lower boil time compared to the stoves dependent upon solid biomass fuels.

Longer cooking times could threaten compliance with alternative stove use in a real-world setting. Furthermore, the increase in cooking time could lead to further exposure if emission reduction does not sufficiently balance the amount of time required to stand over a stove. Factors such as cooking practices and daily activities in different cultures and locations could affect these comparisons.

One possible explanation for the increase in boiling time for the alternative stoves in Study I is placement of the fuel. With the traditional stove, fuel was placed under and central to both burners. For alternative stoves, fuel was placed principally under one burner resulting in the flame being unequally distributed. However, cooking practices and adaptability of the families that use these stoves will determine if the unequal distribution of heat is sufficient for their cooking needs. For an extended discussion on implications for exposure given PM_2.5_ concentrations and boiling time, see the appendix. 

With regards to fuel use, the results are consistent in that all of the stoves manufactured by Envirofit required the same or less amount of fuel relative to the comparison stoves. In Study I, the Envirofit G-series stove did not require more fuel than the traditional stove (*p* = 0.29). Study II demonstrated that MAES and NAES both required less fuel relative to the AMBS-II (*p* < 0.001). The MAES and NAES stoves featured the second burner in a more central location to increase heat transfer efficiency [21]. In regions of fuel scarcity, the amount of fuel required for cooking is a critical feature in understanding the usefulness and acceptability of an alternative stove by families.

This study was conducted in a controlled setting during stove use. Data on cooking practices in Nepal, such as time spent cooking and the preferred number of burners would be helpful in order to better compare to measurements made in other locations. We selected the alternative stoves evaluated in this study to have two pot openings to best imitate the traditional stove used in our study area and cultural preferences. However, the design of these stoves is also quite different from other alternative stoves in Central and South America, so any comparison between our results and previous studies should be made with caution.

The use of non-standardized fuels was a limitation in both studies. For fuels containing multiple sources such as wood and dung, the fuel was weighed before and after the test, however, the proportion of individual components in the mixture (i.e., wood and dung) may have varied. While moisture content was not measured in the fuel sources, we expect that moisture content was similar because fuel sources were sun dried and deemed to be sufficiently dry for use as fuel. Also, in Study I, when fuel was collected, it was used over multiple stoves because the schedule randomized the order of which stoves would be tested. This randomization should balance the moisture content in the fuels used across stoves during testing. Study II did not randomize the order in which stoves were tested. However, fuel was collected in large quantities and then used over multiple stoves—balancing the moisture content across stoves.

We did not collect data in this evaluation that would allow us to estimate the actual PM_2.5_ exposure that would result from the day-to-day use of each of the stoves nor did we evaluate PM_2.5_ concentrations with the alternative stoves during actual meal preparation. The goal of this study was to compare stove performance under conditions that closely represented typical stove use while controlling for as many variables as possible.

## 5. Conclusions

Study I demonstrated that the alternative stoves, AMBS-I and Envirofit G-series, reduced mean PM_2.5_ in comparison with a traditional stove by 60% and 50%, respectively. Accompanying this reduction, boiling time increased by 86% and 104%, respectively, and in the case of AMBS-I, fuel use increased by 39%. If the longer boiling times translate to longer cooking times in homes, there may be implications for end user acceptability and exposure to PM_2.5_. Study II demonstrated how alterations in the G-series stove reduced mean PM_2.5_ relative to the AMBS-II, with MAES and NAES both resulting in 39% reductions. Determining how the results from Studies I and II translate to PM_2.5_ reductions in actual homes, for those families using the alternative stoves, is being assessed in the actual randomized trials.

## Dedication

This manuscript is dedicated to Alison Geyh from the Department of Environmental Health Sciences, Johns Hopkins Bloomberg School of Public Health who passed away before this paper was completed. She was a great friend and outstanding colleague.

## Appendix

### Regarding Nephelometric Particulate Matter Conversion to Gravimetric Equivalent

The following addresses modifications of an equation for the conversion of nephelometric particulate matter concentrations measured at different humidities to PM_2.5_ gravimetric equivalents originally presented in the paper by Soneja *et al.* [24], titled “Humidity and Gravimetric Equivalency Adjustments for Nephelometer-Based Particulate Matter Measurements of Emissions from Solid Biomass Fuel Use in Cookstoves”. Soneja *et al.* [24] provided a humidity and gravimetric conversion equation that is applicable for nephelometric PM concentration ranging from ~600 μg/m^3^ to ~66,000 μg/m^3^. In order to extend the range of this conversion to lower concentrations, additional data were collected during June 2014 in the same test house used in the original paper at PM concentrations below 600 μg/m^3^. The same co-located nephelometric and gravimetric methods were utilized to collect data from 25 simulated cooking tests that followed the same protocol. Data from these new tests were then integrated into the original data set that was used for creation of the Soneja *et al.* conversion equation.

### Updated Findings

This appendix describes updates to figures, equation coefficients, and RMSEs to the original paper presented by Soneja *et al* [24]. After incorporating these 25 new data points into the original dataset, the goodness of fit comparison remained the same as in the original paper. Table A1 shows updated equation coefficients and RMSE for Equation (A1) listed below (which corresponds to Equation (8) from the original Soneja *et al.* paper [24]). Table A2 shows updated RMSEs for all of the conversion approaches outlined in the original paper. The updated RMSEs indicate that use of the combined spline approach (Equation (A1) below) is the best gravimetric equivalency conversion approach, which agrees with findings from the original paper by Soneja *et al.* [24]. Therefore, Equation (A1) from below along with the updated coefficient values, was utilized for conversion of nephelometric PM data to PM_2.5_ gravimetric equivalents.

(A1) $$\text{ln}\left({\text{Gravimetric PM}}_{2.5}\right)=\text{a}+\text{b}\times \text{ln}\left(1-\text{RH}\right)+\text{c}\times \text{ln}\left(\text{Nephelometric PM}\right)+\text{ d}\times \left(\text{ln}\left(\text{Nephelometric PM}\right)-\text{e}\right)$$

**Table A1:** Update of coefficients and RMSE on combined spline adjustment approach.

Equation	Intercept (a)	RH (b)	Nephelometer (c)	Spline (d)	Spline Knot ^1^ (e)	RMSE
C-spline (Equation (8))	0.36	0.69	1.07	−0.43	8.0	2537

^1^ If ln(Nephelometric PM) is <8, then the last term, which is “d × (ln(Nephelometric PM) − e)”, becomes a 0.

**Table A2:** Updates of RMSEs on different overall pDR-1000 adjustment approaches (updated version of original Table 3 from Soneja et al., 2014 [24]).

Quality Control Method Type	RMSE (μg/m^3^)
Combined Approach Equation (1)	3750
Combined Approach—spline Equation (2)	2552
Combined Approach—quadratic Equation (3)	2758
Equations (1*a*), (1*b*) and (2*a*) (without threshold) + Equation (4)	4245, 3928, 3801
Equations (1*a*), (1*b*) and (2*a*) (without threshold) + Equation (5)	2231, 2238, 2169
Equations (1*a*), (1*b*) and (2*a*) (without threshold) + Equation (6)	2280, 2248, 2222
Equations (1*a*), (1*b*) and (2*a*) (with threshold) + Equation (4)	4159, 4338, 4556
Equations (1*a*), (1*b*) and (2*a*) (with threshold) + Equation (5)	2247, 2283, 2304
Equations (1*a*), (1*b*) and (2*a*) (with threshold) + Equation (6)	2298, 2328, 2359

### Regarding Calculation of Exposure to Particulate Matter from Various Stove Use

Study I showed an increase in boiling time for both alternative stoves, Envirofit G and AMBS-I, in comparison with the traditional stove. The increase was highly significant in the multivariate analysis for both stoves (*p* < 0.001). If such an increase in boiling time translates to an increase in cooking time in the homes, people may be exposed to PM_2.5_ for longer periods of time even if those PM_2.5_ concentrations are lower than they would have been with the traditional stove. For this reason, we wanted to calculate potential exposure for Study I and II.

In many examples in the literature, personal exposure to PM_2.5_ from cookstoves is done in actual homes. The family uses the cookstove for their heating and cooking needs and PM_2.5_ concentrations are measured over a defined period of time. For example, in one study in Mexico, personal exposure was measured over 24-hours by two instruments—the University of California at Berkeley (UCB) particle monitor as well as a CO monitor—which were carried in a shoulder bag worn by the participant [33]. In one study in Guatemala, personal exposure of children was measured over 24-hours using a Gastec 1D tube placed on the child’s clothing that measured CO concentrations as a proxy for PM_2.5_ [34]. In another study in Guatemala, personal exposure was measured over 48-hours using Dositubes by Gastec worn by mothers that measured CO as a proxy for exposure to the burning of wood [35].

To create a measure of exposure from Study I and Study II, we multiplied the average PM_2.5_ concentration adjusted for background from each test by the test’s boiling time. We then calculated summary measures for each stove and fuel combination. The results are shown Table A3 and Table A4.

The exposure measurements for Study I for the Envirofit G-series stove are similar, but slightly higher exposures for all fuel types when compared to the traditional stove. For example, the mean exposure using wood for fuel for the traditional stove was 3034 (μg × hrs)/m^3^, but for the Envirofit G-series, the mean exposure was 3445 (μg × hrs)/m^3^. When comparing the AMBS-I stove to the traditional, the mean exposure is lower for AMBS-I for wood and wood/dung, but higher for AMBS-I for crop waste. In Study II, AMBS-II had the highest exposure measurements for all biofuel types when compared with NAES and MAES. For example, the mean exposure using wood for fuel and a closed door/window for AMBS-II was 2450 (μg × hrs)/m^3^, but was 993 and 1807 (μg × hrs)/m^3^ for MAES and NAES, respectively.

**Table A3:** Summary of exposure in Study I comparing two alternative cookstoves to a traditional stove in a home setting in rural Nepal.

Measurement	Fuel	Traditional	*n*	AMBS-I	*n*	Envirofit G-Series	*n*
		Mean (SD)		Mean (SD)		Mean (SD)	
**Exposure ^a^ (μg × hrs)/m^3^**	Wood	3034 (1592)	23	1959 (1087)	12	3445 (1452)	9
	Wood and Dung	4355 (2158)	13	1555 (617)	6	6674 (7177)	9
	Crop Waste	7673 (5237)	12	9947 (6698)	5	8598 (5871)	6

^a^ Exposure was calculated by multiplying the mean PM_2.5_ concentration for each test by the boiling time. Background concentrations were subtracted from mean PM_2.5_ concentrations. Background PM_2.5_ was calculated from measurements taken during the 30-min period before the water boiling test began. Summary measures were created for the stove and fuel combinations in the table.

**Table A4:** Summary of exposure in Study II comparing three alternative cookstoves with an improved brick stove in a home setting in rural Nepal.

Measurement	Fuel	Window/Door Status	AMBS-II	*n*	MAES	*n*	NAES	*n*	LPG	*n*
			Mean (SD)		Mean (SD)		Mean (SD)		Mean (SD)	
**Exposure ^a^ (μg × hrs)/m^3^**	Wood	Closed	2450 (349)	3	993 (102)	2	1807 (1611)	3		
		Open	2408 (1412)	3	986 (154)	3	977 (368)	3		
	Wood/Dung/ Crop waste	Closed	3244 (1750)	3	1632 (2)	2	1973 (741)	3		
		Open	2562 (1728)	3	2479 (1885)	3	2207 (853)	3		
	LPG ^b^	Closed							49 (60)	6
		Open							57 (35)	6

**^a^** Exposure was calculated by multiplying the mean PM_2.5_ concentration for each test by the boiling time. Background concentrations were subtracted from mean PM_2.5_ concentrations. Background PM_2.5_ was calculated from measurements taken during the 30-min period before the water boiling test began. Summary measures were created for the stove and fuel combinations in the table; ^b^ PM_2.5_ concentrations were not adjusted for background levels due to resultant negative values.

In the literature, exposure measurements in the form of PM_2.5_ concentrations were collected over a set period of time, such as 24-hours [33,34]. The measurements were made in actual homes where a family was using the cookstoves [33,34,35]. In contrast, in Study I and Study II, instead of collecting PM_2.5_ concentrations over a uniform period of time, PM_2.5_ concentrations were collected over whatever the length of time was necessary to boil 20 L of water for Study I and 10 L of water for Study II. Our study was not in an actual home where meals were being prepared, but was in a test house where controlled water boiling tests were being conducted. Individual cooking practices in actual homes will influence exposure. Study I and II did not take into account the variety of cooking practices and needs. Depending on the cooking needs of the family, the use of the different burners will vary. One of the factors which may have influenced boiling times in Study I, described in the discussion, was the placement of fuels under the pot openings. For the traditional stove, fuel was placed under the middle of the two pot openings. For the alternative cookstoves, fuel was placed under one pot opening. In our experience, the burner directly above the fuel boiled the water faster. If families mostly use this burner, their cooking times may be shorter than those families who are trying to cook using both burners on the alternative stoves. For these reasons, any exposure results from Study I and II must be interpreted with caution. The NCT will be measuring environmental conditions including PM_2.5_ over a 24 h time period, and will provide a more accurate measure of exposure than was collected in Study I and Study II.
